# Temporal shifts in prognostic factors for 90- and 180-day outcomes after stroke thrombolysis: A machine learning analysis

**DOI:** 10.1371/journal.pone.0338011

**Published:** 2025-12-04

**Authors:** Aixia Chen, Ao Li, Youli Jiang, Huan Ye

**Affiliations:** 1 Department of Neurology, People’s Hospital of Longhua, Shenzhen, China; 2 Clinical Nursing Teaching and Research Section, The Second Xiangya Hospital of Central South University, Changsha, China; UCSF: University of California San Francisco, UNITED STATES OF AMERICA

## Abstract

**Introduction:**

Prognostication at 90 and 180 days after thrombolysis for acute ischemic stroke (AIS) is critical, yet the temporal evolution of key predictors remains inadequately understood. The utility of machine learning for systematically comparing prognostic factors across these distinct time points remains to be fully established.

**Method:**

We retrospectively analyzed consecutive AIS patients undergoing intravenous thrombolysis from October 2020 to December 2024. Features were selected from pre-therapy baseline data via univariable analysis and LASSO regression to develop five machine learning models. Model performance and clinical utility were assessed by AUC-ROC and Decision Curve Analysis (DCA), respectively. The primary endpoint was a modified Rankin Scale > 2 at 90 and 180 days.

**Results:**

A total of 432 patients were included. At 90 days, 81 patients (18.8%) had an unfavorable outcome. By the 180-day follow-up, 86 patients (19.9%) were lost to follow-up. Among the remaining 346 patients, 48 (13.9%) had an unfavorable outcome. On the holdout test set, Logistic Regression (AUC = 0.757) and Random Forest (AUC = 0.833) were the optimal models for 90- and 180-day outcomes, respectively. While baseline NIHSS score and age were dominant predictors for both endpoints, a notable temporal shift in biomarker significance emerged: admission fibrinogen was a key predictor at 90 days, but was supplanted by white blood cell count for the 180-day prognosis.

**Conclusion:**

Our study reveals a crucial temporal evolution in prognostic biomarkers after thrombolysis, shifting from fibrinogen at 90 days to white blood cell count at 180 days. This dynamic landscape of predictors, identified through machine learning, underscores the necessity of developing time-specific models to accurately forecast patient recovery.

## Introduction

Acute ischemic stroke (AIS) is the most common subtype, accounting for 72.9% of all stroke cases in China. As a leading cause of mortality and disability in adults, it places a significant burden on families and society [1,2]. Intravenous thrombolysis, when administered within the therapeutic window, is the standard of care for salvaging the ischemic penumbra and improving functional outcomes [3]. However, clinical outcomes after thrombolysis vary widely. One major challenge is futile recanalization—the failure to achieve good long-term functional recovery despite successful vessel reopening. This phenomenon highlights the critical need for accurate early prognostication [4].

In stroke research, the 90-day functional outcome, measured by the modified Rankin Scale (mRS), is the gold-standard endpoint for assessing therapeutic efficacy [5]. However, since neurological function can continue to improve beyond this time point, the 180-day outcome may better reflect a patient’s stable, long-term functional status [6]. Evaluating both endpoints offers a more complete picture of the patient’s recovery trajectory and can help guide stage-specific rehabilitation strategies. Nevertheless, likely due to the challenges of extended follow-up, the 180-day outcome has been studied less frequently, which limits our understanding of the full long-term benefits of thrombolytic therapy.

To better predict functional outcomes after thrombolysis for AIS, previous research has identified key factors associated with an unfavorable 90-day prognosis [7,8]. These factors span multiple domains, including baseline characteristics, acute clinical metrics, and laboratory biomarkers. At baseline, advanced age, a history of prior stroke, and pre-existing functional disability (mRS > 1) are established risk factors [7,9]. Among core clinical features, the severity of neurological deficit on admission, as measured by the National Institutes of Health Stroke Scale (NIHSS) score, is considered the most potent independent predictor, while onset-to-treatment time (ONT), glucose levels, and blood pressure also play important roles [8]. Furthermore, specific laboratory biomarkers, such as white blood cell count, fibrinogen, and neutrophils, have also been linked to 90-day functional outcomes [10–12]. However, it remains unclear if these factors are also predictive of 180-day outcomes or if their relative importance changes over this longer period. Therefore, this study aimed to develop and validate a predictive model for 180-day functional outcomes to determine the long-term prognostic value of these key factors.

In recent years, machine learning (ML) algorithms have become powerful tools for building precise prognostic models. Their strength lies in their ability to capture complex non-linear relationships and handle high-dimensional data [13]. In stroke research, ML models have shown superior performance compared to traditional statistical models for predicting complications like hemorrhagic transformation (HT) [14] and early neurological deterioration (END) [15]. However, ML techniques have been less commonly applied to predict mid- to long-term functional outcomes after thrombolysis [16]. This gap exists for two main reasons. First, research on 180-day prognosis is scarce. The only modeling study we identified was limited for early clinical decision-making because it included “length of hospital stay” as a predictor [17]. Second, few studies have systematically compared the performance of different ML models for predicting both 90- and 180-day outcomes in the same patient cohort.

Therefore, this study aims to develop and systematically compare several machine learning models using clinical data from a cohort of AIS patients who received intravenous thrombolysis. Our primary objectives are to identify the best-performing models for predicting unfavorable outcomes at 90 and 180 days and to determine the key clinical predictors at each of these time points. We expect this research to provide clinicians with accurate prognostic tools for different post-treatment stages and to offer new evidence on the long-term benefits of thrombolytic therapy.

## Materials and methods

This study follows the Transparent Reporting of a multivariable prediction model for Individual Prognosis Or Diagnosis (TRIPOD) guidelines [18].

### Study participants

This was a retrospective analysis of prospectively collected data from consecutive patients with AIS who received IVT with recombinant tissue plasminogen activator (rt-PA) at People’s Hospital of Longhua, Shenzhen between October 2020 and December 2024. The hospital’s Department of Neurology is a major stroke center serving a population of approximately 3 million. All patients were treated in the emergency observation room following standardized protocols.

Inclusion criteria were as follows: (1) were aged ≥18 years; (2) met the diagnostic criteria for AIS and indications for IVT according to the 2018 Guidelines for the Early Management of Patients with Acute Ischemic Stroke developed by the American Heart Association (AHA)/American Stroke Association (ASA); (3) received rt-PA (alteplase; total dose 0.9 or 0.6 mg/kg; maximum dose 90 mg) within 4.5 hours after symptom onset; (4) had a premorbid mRS score ≤1; and (5) had complete clinical, laboratory, and follow-up data available at 90 days. We excluded patients who: (1) underwent concurrent intra-arterial thrombectomy; (2) had a pre-existing functional disability (premorbid mRS > 1); or (3) had a posterior circulation infarction. This study was approved by the Institutional Review Board of the People’s Hospital of Longhua, Shenzhen (Approval No. 2024073). Data were accessed for research on January 15, 2025. All patient data were fully anonymized prior to analysis, and the authors had no access to identifying information. For the secondary analysis of 180-day outcomes, follow-up data were available for 346 of the 432 eligible patients (80.1%).

### Data collection

We collected the following baseline data for each patient prior to IVT through standardized assessments. Clinical parameters included demographic characteristics (age, sex), vascular risk factors (hypertension, diabetes mellitus, atrial fibrillation, coronary artery disease, prior stroke, smoking status, alcohol consumption), baseline functional status (premorbid mRS score), stroke severity (NIHSS score), TOAST classification, and critical time intervals (onset-to-door time, door-to-needle time). Laboratory parameters included (LDL cholesterol), coagulation studies (fibrinogen, international normalized ratio, D-dimer), inflammatory markers (high-sensitivity C-reactive protein), hematological parameters (white blood cell count), and metabolic indices (blood glucose, serum creatinine). All laboratory tests were performed prior to IVT using standardized automated analyzers. Fibrinogen levels were measured using the Clauss method. Attending neurologists and emergency physicians documented the clinical data, which were then entered into a specialized research database by trained personnel. The database underwent regular quality control assessments. Missing data were imputed using the K-Nearest Neighbors (KNN) method. To ensure model reliability, variables with >10% missing values were excluded from the analysis.

### Clinical outcomes

Trained neurologists, blinded to the study objectives, assessed clinical outcomes at 90 days for all participants through structured telephone interviews or outpatient follow-up visits. The same procedure was followed for the 180-day assessment, for which data were available for 346 participants. Functional outcomes were evaluated using the mRS. A mRS score of ≤2 indicated functional independence (good outcome), whereas a mRS score of >2 suggested functional dependence (unfavorable outcome).

### Sample size justification

Sample size adequacy was retrospectively assessed using the method proposed by Riley et al. for prediction model development [19]. Based on an expected C-statistic of 0.70 and acceptable margin of error of 0.05, the minimum required sample size was calculated as:


$$n=\frac{(1.96{)}^{2}\times 0.188\times 0.812}{(0.05{)}^{2}}\approx 233$$ (1)

Our cohort of 432 patients exceeded this minimum requirement, providing sufficient statistical power for model development and validation.

### Statistical analysis

All statistical analyses were conducted in Python (version 3.10) using key libraries such as NumPy, Pandas, SciPy, Statsmodels, and Scikit-learn. Continuous variables are expressed as mean ± standard deviation (SD) for normally distributed data and median [interquartile range (IQR)] for non-normally distributed data. Categorical variables were presented as counts and percentages (%). The Shapiro-Wilk test was used to assess the normality of continuous variables. For baseline comparisons, independent samples t-tests or Mann-Whitney U tests were used for continuous variables as appropriate. Chi-square or Fisher’s exact tests were used for categorical variables, with the latter applied when expected cell counts were below 5. For modeling, categorical variables were one-hot encoded, and continuous variables were standardized using z-score normalization. A two-stage feature selection process was employed to identify predictors for the primary functional outcome (defined as mRS 0–2 at 90 days). First, univariate analysis was conducted, and variables with a p-value < 0.10 were considered potential predictors. Second, a Least Absolute Shrinkage and Selection Operator (LASSO) regression model with 5-fold cross-validation was applied to these candidate variables to perform final feature selection and mitigate overfitting. A p-value < 0.05 was considered statistically significant for all final analyses.

To assess potential selection bias due to the 19.9% loss to follow-up at 180 days, we compared baseline characteristics between patients with (n = 346) and without (n = 86) follow-up data. The groups were similar in most key variables, including NIHSS score (p = 0.76), though small vessel occlusion was less frequent in the lost-to-follow-up group (p = 0.005) (S1 Table). Stratifying by NIHSS quartiles confirmed no association between stroke severity and follow-up status (p = 0.316) (S2 Table). Finally, a sensitivity analysis imputing outcomes under worst- and best-case scenarios established a plausible range for the true 180-day outcome rate (S3 Table).

### Model development

We trained and evaluated five machine learning algorithms to predict the primary functional outcome: Logistic Regression (LR), Random Forest (RF), Decision Tree (DT), SVM, and Extreme Gradient Boosting (XGBoost). The dataset was randomly partitioned into a training set (80%) and a holdout testing set (20%). Stratified sampling based on the outcome variable was used to ensure proportional representation in both sets. Hyperparameters for each model were tuned on the training set using a 5-fold cross-validation grid search (GridSearchCV). Using the optimized hyperparameters, the models were then retrained on the entire training set. Final model performance was evaluated on the holdout testing set. Evaluation metrics included the Area Under the Receiver Operating Characteristic Curve (AUC-ROC), precision, recall, F1-score, and accuracy. The DeLong test was used to statistically compare the AUCs of different models. The RF model, which demonstrated the best performance (see Results), was selected for all subsequent analyses. To compare the evolution of predictor importance over time, we analyzed the feature importance profiles of the RF models trained for 90-day and 180-day outcomes. Finally, a nomogram was created for clinical application, and patients were stratified into risk groups based on the predicted probabilities from the 180-day RF model.

## Results

### Patient characteristics

**Fig 1:**
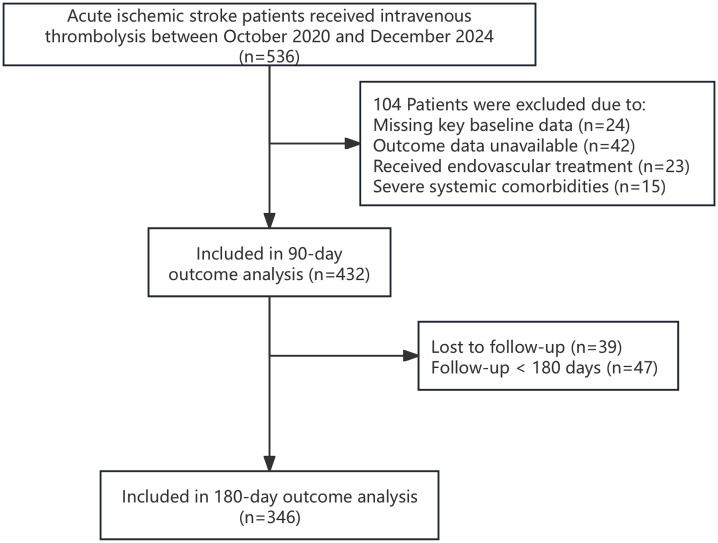
Flowchart of Patient Enrollment and Selection.

We initially screened 536 patients with acute ischemic stroke who received intravenous thrombolysis between October 2020 and December 2024 were initially identified. As detailed in the flowchart (Fig 1), 104 patients were excluded due to missing key baseline data (n = 24), unavailable outcome data (n = 42), receipt of endovascular treatment (n = 23), or severe systemic comorbidities (n = 15), leaving a final cohort of 432 patients for the 90-day outcome analysis.

Of these 432 patients, 81 (18.8%) experienced an unfavorable outcome (mRS > 2) at 90 days. For the 180-day analysis, 86 patients were excluded due to loss to follow-up (n = 39) or incomplete follow-up (<180 days; n = 47), leaving 346 patients for the long-term analysis. Among these, 48 (13.9%) had an unfavorable outcome. Baseline demographic and clinical characteristics of the cohort are shown in Table 1.

**Table 1:** Baseline Demographic and Clinical Characteristics of the Study Cohort.

Characteristic	Total (N = 432)	90 Days Outcome		P-Value	180Days Outcome		P-Value
		Good Outcome (n = 351)	Unfavorable Outcome (n = 81)		Good Outcome (n = 298)	Unfavorable Outcome (n = 48)	
**Demographics**							
Age, years	57.0 [48.0-68.0]	56.0 [47.0-66.5]	66.0 [56.0-77.0]	<0.001	56.0 [47.0-67.0]	65.0 [54.0-79.2]	<0.001
Male sex	309 (71.5)	255 (72.6)	54 (66.7)	0.350	217 (72.8)	32 (66.7)	0.570
Alcohol consumption	89 (20.6)	77 (21.9)	12 (14.8)	0.200	73 (24.4)	6 (12.5)	0.200
Current or former smoking	152 (35.2)	128 (36.5)	24 (29.6)	0.300	117 (39.2)	11 (22.9)	0.070
**Baseline Assessment**							
Premorbid mRS score	0.0 [0.0-1.0]	0.0 [0.0-1.0]	0.0 [0.0-2.0]	0.090	0.0 [0.0-1.0]	0.0 [0.0-2.2]	0.002
Pre-treatment NIHSS score	4.0 [2.0-8.0]	4.0 [2.0-7.0]	9.0 [4.0-14.0]	<0.001	4.0 [2.0-7.0]	11.0 [5.8-14.2]	<0.001
Baseline GCS score	15.0 [15.0-15.0]	15.0 [15.0-15.0]	15.0 [12.0-15.0]	<0.001	15.0 [15.0-15.0]	15.0 [12.0-15.0]	<0.001
**Medical History**							
Hypertension	139 (32.2)	118 (33.6)	21 (25.9)	0.230	93 (32.3)	12 (25.0)	0.400
Diabetes mellitus	70 (16.2)	51 (14.5)	19 (23.5)	0.070	47 (16.3)	8 (16.7)	1.000
Atrial fibrillation	18 (4.2)	12 (3.4)	6 (7.4)	0.120	9 (3.1)	5 (10.4)	0.040
Coronary artery disease	15 (3.5)	14 (4.0)	1 (1.2)	0.320	12 (4.2)	0 (0.0)	0.230
Prior stroke	71 (16.4)	58 (16.5)	13 (16.0)	1.000	49 (17.0)	8 (16.7)	1.000
Prior myocardial infarction	2 (0.5)	2 (0.6)	0 (0.0)	1.000	2 (0.7)	0 (0.0)	1.000
Prior atrial fibrillation	17 (3.9)	11 (3.1)	6 (7.4)	0.110	7 (2.4)	5 (10.4)	0.020
Prior coronary artery disease	24 (5.6)	17 (4.8)	7 (8.6)	0.180	12 (4.2)	4 (8.3)	0.260
**TOAST Classification**							
Large artery atherosclerosis	162 (37.5)	118 (33.6)	44 (54.3)	<0.001	100 (33.5)	27 (56.2)	0.001
Small vessel occlusion	176 (40.7)	154 (43.9)	22 (27.2)	0.008	139 (48.3)	10 (20.8)	<0.001
Cardioembolism	33 (7.6)	25 (7.1)	8 (9.9)	0.540	18 (6.2)	8 (16.7)	0.020
Other determined	31 (7.2)	26 (7.4)	5 (6.2)	0.880	18 (6.2)	2 (4.2)	0.750
Undetermined	30 (6.9)	28 (8.0)	2 (2.5)	0.130	23 (8.0)	1 (2.1)	0.220
**Time Metrics**							
Onset to door time, minutes	90.5 [51.0-150.0]	90.0 [52.0-150.5]	102.0 [50.0-145.0]	0.790	96.0 [55.0-151.0]	77.0 [38.5-140.0]	0.070
Door to needle time, minutes	36.0 [24.0-32.0]	36.0 [24.0-32.0]	36.0 [24.0-32.0]	0.910	36.0 [24.0-32.0]	36.0 [24.0-36.0]	0.660
**Laboratory Values**							
LDL cholesterol, mmol/L	3.0 [2.4-3.6]	3.0 [2.4-3.6]	3.0 [2.4-3.6]	0.940	2.9 [2.4-3.6]	3.0 [2.3-3.5]	0.890
Fibrinogen, g/L	2.9 [2.4-3.5]	2.9 [2.4-3.4]	3.1 [2.6-3.7]	0.030	2.9 [2.4-3.4]	3.1 [2.6-4.1]	0.100
White blood cell count, × 10⁹/L	8.0 [6.5-9.6]	7.8 [6.5-9.4]	8.6 [6.8-10.2]	0.040	8.0 [6.5-9.3]	9.2 [7.5-11.2]	0.003
High-sensitivity C-reactive protein, mg/L	0.6 [0.5-2.9]	0.5 [0.5-2.5]	1.0 [0.5-5.8]	0.003	0.5 [0.5-2.5]	0.9 [0.5-5.2]	0.090
International normalized ratio	1.0 [0.9-1.0]	1.0 [0.9-1.0]	1.0 [0.9-1.1]	0.020	1.0 [0.9-1.0]	1.0 [0.9-1.1]	0.060
D-dimer, mg/L	0.3 [0.2-1.1]	0.3 [0.2-1.0]	0.4 [0.2-1.5]	0.140	0.3 [0.2-0.8]	0.5 [0.2-1.4]	0.060
Blood glucose, mmol/L	5.5 [5.1-6.8]	5.4 [5.0-6.5]	5.7 [5.2-9.0]	0.002	5.3 [5.0-6.6]	5.7 [5.2-7.4]	0.020
Serum creatinine, μmol/L	77.0 [63.0-91.6]	76.8 [62.7-91.0]	78.6 [65.0-97.0]	0.500	76.0 [62.0-91.0]	80.7 [65.2-100.2]	0.130

### Univariate analysis and LASSO feature selection

Following this, variables with a P-value < 0.10 were entered into a LASSO regression model unfavorable outcomes at both 90 and 180 days, including older age, higher pre-treatment NIHSS score, and large-artery atherosclerosis subtype (all P < 0.1, S4 Table). Conversely, the small-vessel occlusion subtype was a consistent predictor of a good outcome.

**Fig 2:**
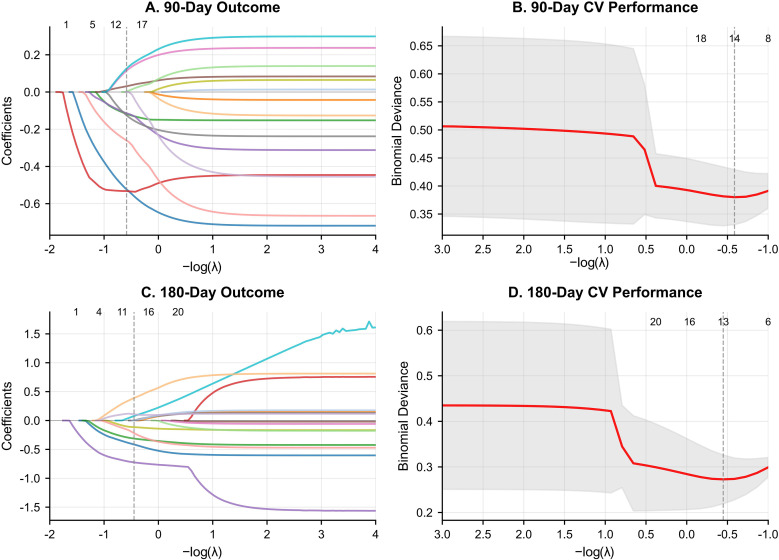
Feature Selection Using LASSO Regression for Predicting 90-Day and 180-Day Outcomes.

Variables with a P-value < 0.10 were subsequently included in a LASSO regression model to mitigate multicollinearity and select the most robust predictors. The optimal penalty parameter (λ) was selected by minimizing the binomial deviance, as shown in Fig 2. This process yielded 14 predictors for the 90-day model and 13 for the 180-day model. The pre-treatment NIHSS score and age were the most influential predictors for the 90-day outcome, while the NIHSS score was the single most powerful predictor for the 180-day outcome (S5 Table).

### Machine learning model performance and clinical utility

**Fig 3:**
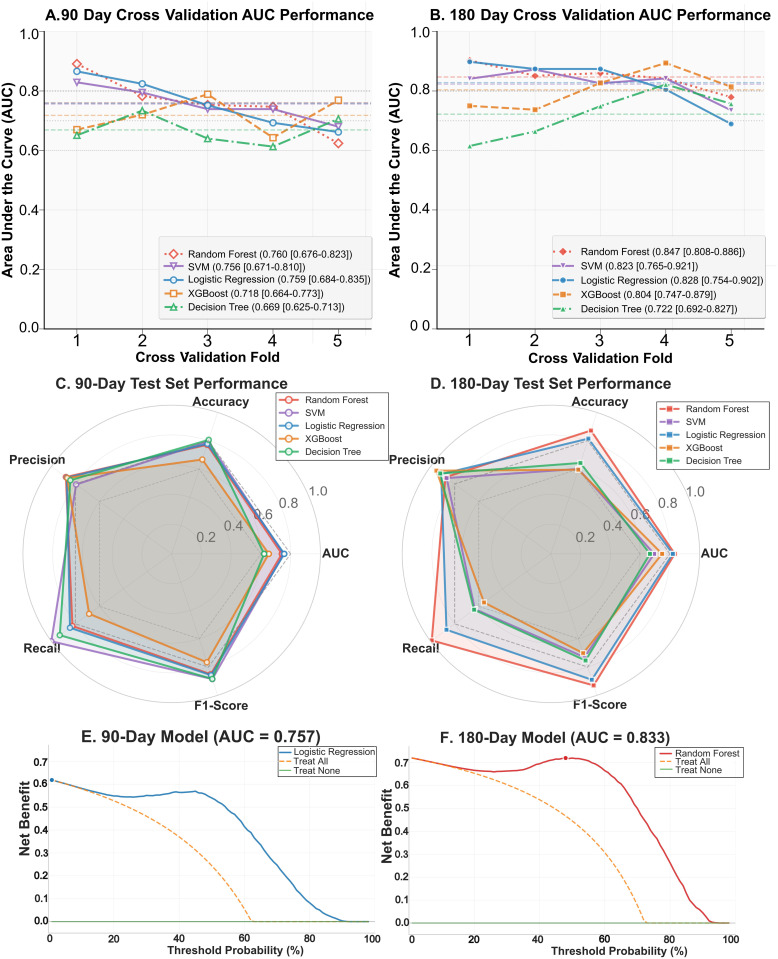
Comparative Performance of Models Showing (A, B) Cross-Validation AUC, (C, D) Test Set Radar Plots, and (E, F) Decision Curve Analysis.

In 5-fold cross-validation, the RF model demonstrated the highest mean Area Under the Curve (AUC) for the 90-day endpoint (mean AUC = 0.760, 95% CI [0.676–0.823]), closely followed by the Logistic Regression (mean AUC = 0.759, 95% CI [0.684–0.835]) and SVM (mean AUC = 0.756, 95% CI [0.671–0.810]) models (Fig 3A). For the 180-day endpoint, the RF model again achieved the highest performance (mean AUC = 0.847, 95% CI [0.808–0.886]), followed by the Logistic Regression (mean AUC = 0.828, 95% CI [0.754–0.902]) and SVM (mean AUC = 0.823, 95% CI [0.765–0.921]) models (Fig 3B). The superior performance of these models was confirmed on the holdout test set, with radar plots showing balanced performance across accuracy, precision, recall, and F1-score (Fig 3C, 3D).

To evaluate the clinical utility of the models, we performed Decision Curve Analysis (DCA). The analysis showed that the top-performing 90-day LR model (Test set AUC = 0.757) and 180-day RF model (Test set AUC = 0.833) also yielded the highest net benefit across a wide range of clinically relevant threshold probabilities (Fig 3E, 3F). This indicates that using these models to inform clinical decisions would be more beneficial than the default strategies of treating all or no patients, thereby confirming their clinical utility.

### Feature importance in predicting outcomes

**Fig 4:**
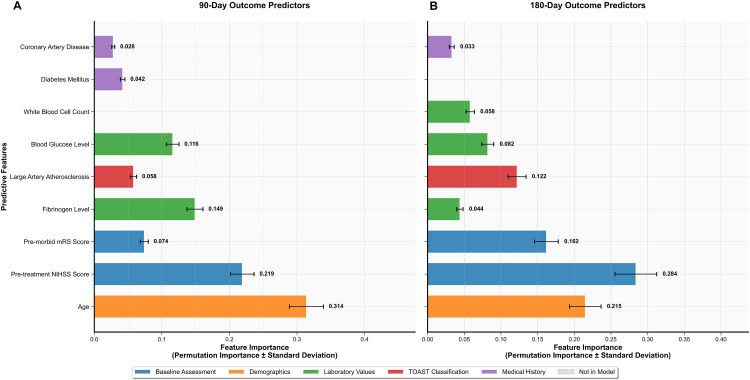
Feature Importance Analysis: Random Forest Models for Stroke Outcome Prediction Temporal Evolution from 90-Day to 180-Day Outcomes.

To investigate the temporal evolution of predictor importance, we compared feature importance from the RF models for 90- and 180-day outcomes (Fig 4). To highlight temporal shifts, features in the plot are unified and sorted by descending importance for the 180-day outcome. While Pre-treatment NIHSS Score and Age are confirmed as the two most dominant predictors across both time points, reinforcing their fundamental role, the analysis revealed a significant prognostic shift. Acute physiological markers, most notably Fibrinogen and Blood Glucose, were highly influential for the 90-day outcome, but their predictive power diminished substantially when predicting the 180-day outcome. Conversely, the importance of factors representing underlying patient state and potential chronic inflammation, such as White Blood Cell Count and Pre-morbid mRS score, became more pronounced over the longer term. This finding demonstrates an evolution from the influence of acute-phase physiological factors in short-term prediction to the greater importance of baseline patient characteristics and chronic inflammatory states for long-term prognosis.

## Discussion

In this study, we developed and validated multiple machine learning models to predict long-term unfavorable outcomes in patients with AIS following intravenous thrombolysis. Our results indicate that the models demonstrated good to excellent discriminative performance. The optimal model, however, varied by the prediction endpoint: LR demonstrated the best overall performance for 90-day outcomes, whereas RF proved superior for 180-day predictions. Our analysis first confirmed the importance of established predictors, particularly the pre-treatment NIHSS score and patient age, which are fundamental to prognostic assessment. Notably, our findings revealed a dynamic shift in the predictive importance of key biomarkers when predicting outcomes at the 90-day versus the 180-day time point. This finding suggests evolving pathophysiological mechanisms that characterize distinct phases of post-stroke recovery.

A key finding of this study is the dynamic shift in the prognostic importance of different biomarkers over time following thrombolysis. The biomarkers analyzed, including fibrinogen and white blood cell count, were derived from the first laboratory tests conducted within 24 hours of admission, providing a critical snapshot of the pathophysiological state during the hyperacute phase of stroke. For 90-day outcomes, the admission fibrinogen level was a strong predictor. This is not only because fibrinogen, as an acute-phase reactant, reflects the immediate post-stroke inflammatory storm and blood-brain barrier disruption, but also because its significance is directly linked to the core therapeutic intervention of intravenous thrombolysis [20]. Elevated fibrinogen levels may signify a greater initial thrombus burden or potential resistance to thrombolytic therapy [21]. These factors are decisive for early neurological outcomes. This observation is consistent with previous studies, which have repeatedly reported a strong association between fibrinogen levels and 90-day prognosis in patients with AIS, both with and without thrombolysis [22]. However, when the observational window was extended to 180 days, the predictive power of fibrinogen diminished, while the baseline white blood cell (WBC) count emerged as a more important factor for long-term prognosis. This transition strongly suggests that the key pathophysiological drivers shaping the long-term recovery trajectory shift from acute-phase thrombosis and injury to sustained inflammatory and immunomodulatory processes in the subacute and chronic stages [23]. As a sensitive indicator of systemic inflammation, the sustained impact of WBC count may modulate the ultimate outcome over a longer time horizon by influencing neuroimmune responses, affecting neuroplasticity, and potentially increasing the risk of secondary infection [24]. This “temporal drift” in the prognostic value of biomarkers, observed in our cohort of thrombolysis-treated patients with AIS, provides new clues for individualized prognostication in this field. A key avenue for future research is to explore how this time-dependent predictive pattern can be leveraged. For instance, developing novel dynamic models that integrate the prognostic weight of fibrinogen at 90 days and WBC count for long-term prediction could significantly enhance the accuracy of risk stratification, thereby helping to match patients at different recovery stages with the most appropriate rehabilitation and follow-up strategies.

Our 180-day RF model (test set AUC = 0.833) compares favorably with previous prognostic tools, such as a nomogram study reporting an accuracy of 71.99% [17]. We attribute this improved performance to two key factors. First, the RF algorithm can effectively capture complex, non-linear interactions between predictors (e.g., WBC count, age, and glucose), which may better reflect the underlying pathophysiology of stroke [25]. Second, our model relies exclusively on routine laboratory tests available upon admission, ensuring it can be integrated into clinical workflows without additional cost or delay. This contrasts with models requiring advanced imaging or genomic data, which are often impractical in the hyperacute setting.

The observed temporal shift in predictor importance likely reflects the evolving pathophysiology of stroke recovery. Fibrinogen, a key acute-phase reactant, serves as a marker for the initial vascular event and subsequent inflammatory storm. Its elevated levels may indicate a larger thrombus burden or resistance to thrombolysis, and its degradation products can amplify neuroinflammation and blood-brain barrier disruption [26]. Consequently, its predictive power is most pronounced in the early phase (i.e., 90 days). In contrast, the growing importance of the WBC count at 180 days points to the role of sustained systemic inflammation in long-term outcomes. A persistently elevated WBC count may signal unresolved chronic inflammation or stroke-induced immunosuppression, which impairs neurorepair and increases susceptibility to secondary infections—a major cause of long-term morbidity and mortality [27,28]. Therefore, WBC count may act as an early indicator of a patient’s long-term inflammatory burden and resilience, with its impact accumulating over time.

Finally, DCA confirmed the clinical utility of our models. The analysis showed that using the models for decision-making yields a higher net benefit than treating all or no patients across a wide range of risk thresholds. This suggests that the models can serve as practical decision-support tools. For example, by inputting several baseline metrics, clinicians could stratify patients by their long-term risk. Such early risk stratification is crucial for personalizing post-acute care, such as tailoring rehabilitation intensity or scheduling more frequent follow-ups, thereby optimizing resource allocation for patients at highest risk.

Pre-morbid functional status, as measured by the mRS, is an independent and significant predictor of long-term functional outcomes. Its consistent incorporation as a core variable in both conventional multivariable predictive models and advanced machine learning algorithms supports this association [29]. The clinical implication of this finding is that the pre-morbid mRS serves not only as a baseline for prognostic assessment but also as a guide for clinical practice. It enables clinicians to establish realistic rehabilitation expectations with patients and their families early on and to devise more tailored long-term treatment and care plans accordingly. Large artery atherosclerosis (LAA) was identified as an independent predictor of unfavorable prognosis. This observation aligns with the established view that different stroke subtypes follow distinct clinical trajectories and carry varying risks of recurrence [30]. Previous research has consistently demonstrated that strokes secondary to LAA, such as those caused by intracranial arterial stenosis, are associated with poorer long-term outcomes compared to those from small-vessel disease, like lacunar infarcts [31]. Therefore, precise etiological subtyping of stroke, particularly the identification of LAA, is essential for guiding secondary prevention strategies. Decisions regarding whether to intensify statin and antiplatelet therapy or to consider endovascular intervention are important for improving patients’ long-term quality of life, as these interventions target the pathophysiological mechanisms of atherosclerosis. Traditional vascular comorbidities, particularly diabetes mellitus (DM) and coronary artery disease (CAD), were also confirmed as key negative prognostic factors, a conclusion widely substantiated in the literature. Studies have consistently shown that the presence of diabetes significantly worsens functional outcomes in patients with ischemic stroke, both in the acute phase and over the long term [32]. This adverse impact likely reflects a more extensive, underlying systemic vascular pathology. For instance, the combination of hypertension and diabetes not only directly exacerbates unfavorable post-stroke outcomes but also frequently amplifies the risk of future cardiocerebrovascular events through intermediary mechanisms, such as the impairment of renal function [33]. From a clinical standpoint, therefore, a history of DM or CAD should be viewed as an indicator of poor overall vascular health. This underscores the necessity for comprehensive and intensified secondary prevention strategies in these patients, focusing on the systemic management of all coexisting vascular risks rather than solely addressing the index cerebrovascular event.

Several limitations of this study should be acknowledged. First, this was a single-center, retrospective study, which may limit the generalizability of our findings. Although our study included a large sample from Shenzhen, a diverse metropolis, which may support its external validity, the generalizability of our findings may still be limited, and the conclusions require cautious interpretation. Institution-specific protocols and patient demographics might have influenced the results. Therefore, future large-scale, multi-center prospective studies are warranted to validate our models in different populations and healthcare settings. Second, our predictive models were constructed based on clinical and laboratory variables readily available at baseline, but did not include imaging data, such as infarct volume, collateral circulation status, or recanalization grades. Due to the retrospective design, standardizing and retrieving quantitative imaging metrics across all patients. These imaging markers are known to be powerful prognostic factors for stroke outcomes. Future research should aim to integrate multi-modal imaging information to develop more comprehensive and accurate predictive models.

## Conclusion

In this cohort study of patients with acute ischemic stroke receiving intravenous thrombolysis, machine learning models, particularly logistic regression for 90-day and random forest for 180-day outcomes, effectively predicted long-term functional dependence. While confirming the strong predictive power of baseline NIHSS score and age, this study identified a temporal shift in the prognostic significance of key biomarkers. Admission fibrinogen levels were a stronger predictor of 90-day outcomes, whereas white blood cell count emerged as more influential for 180-day prognosis. This finding suggests that the underlying pathophysiological drivers of recovery evolve from acute-phase thrombosis and injury to more sustained systemic inflammatory processes over time. These findings support the use of time-specific machine learning models as practical tools for improving early, individualized prognostication and suggest that tailoring long-term management strategies to the evolving inflammatory state may be important for improving patient outcomes.

## Supporting information

S1 Table Baseline characteristics of patients stratified by 180-day follow-up status. (CSV)

S2 Table Association between baseline stroke severity and loss to follow-up. (XLSX)

S3 Table Sensitivity analysis of the primary outcome under different assumptions for missing data. (CSV)

S4 Table Univariate Analysis Results for 90-Day and 180-Day Outcomes. (CSV)

S5 Table LASSO Regression Variable Selection Results for 90- and 180-Day Prognosis. (XLSX)
